# Editorial: Irritable bowel syndrome: what is known and what is missing in daily practice

**DOI:** 10.3389/fmed.2023.1247319

**Published:** 2023-07-14

**Authors:** Khaled Jadallah, Roberto De Giorgio, David S. Sanders

**Affiliations:** ^1^Department of Internal Medicine, Faculty of Medicine, King Abdullah University Hospital, Jordan University of Science and Technology, Irbid, Jordan; ^2^Department of Translational Sciences, University of Ferrara, Ferrara, Italy; ^3^Department of Infection, Immunity and Cardiovascular Disease, University of Sheffield, Sheffield, United Kingdom

**Keywords:** irritable bowel syndrome, pathophysiology, Rome IV criteria, multidisciplinary, gut microbiota, FODMAPs, clinical practice, brain-gut axis

Irritable bowel syndrome (IBS) is a highly prevalent and burdensome disorder of gut-brain interaction (DGBI) across all ages and ethnicities, and yet, its pathogenesis, diagnosis, and treatment remain elusive (1).

This Research Topic aims to improve our understanding of IBS pathophysiology, diagnostic approach, and treatment options. Our call resulted in eight seminal papers spanning IBS pathophysiology, diagnostics, and treatment modalities. Despite the unquestionable diagnostic aid the Rome IV criteria provide, IBS patient management still generates considerable direct and indirect costs for healthcare systems worldwide. Therefore, we deemed it crucial to elucidate the potential factors influencing IBS for better diagnosis and treatment.

In their survey, Nilsson and Ohlsson investigated sociodemographic and lifestyle factors associated with gastrointestinal (GI) symptoms and IBS showing that female sex, smoking, and unemployment in men were significant correlates. Conversely, GI symptoms were inversely associated with higher age and intermediate physical activity. Raising awareness of the modifiable risk factors and lifestyle adjustments is expected to decrease the rate and severity of IBS and GI symptoms in the general population.

Lindberg and Mohammadian brilliant perspective article explored the differential diagnosis and disease associations with IBS. The authors underscored the importance of considering autism spectrum disorders and connective tissue disorders (namely Ehlers-Danlos syndrome or hypermobility disorders) when dealing with difficult-to-treat IBS patients. Patients re-referred to tertiary care for persistent IBS-like symptoms should be re-investigated, considering the broad spectrum of IBS-like disorders and IBS-associated diseases.

Despite extensive research focusing on IBS and the contribution provided by the Rome criteria, definitive tests to positively diagnose this disorder still need to be developed. In daily clinical practice, the diagnosis of IBS remains one of exclusion because of the lack of biomarkers that differentiate and categorize IBS patients (2). The study by Van Malderen et al. analyzed breath and fecal samples from IBS patients and healthy controls via multi-capillary column/ion mobility spectrometry, and classification models were created based on volatile organic compounds (VOCs) and clinical characteristics. Breath and fecal VOCs could diagnose and subtype IBS compared to healthy controls. Yet, additional research is needed to clarify further the relationship between the gut microbiota on the one hand and its manipulation using pharmacologic and non-pharmacologic and VOCs profiles on the other hand.

Over the past decades, several pharmacologic and non-pharmacologic IBS treatment options have been proposed, but the efficacy has been disappointing, with patient dissatisfaction and increased healthcare expenditure (3, 4). In recent years, FODMAP-restricted diet and fecal microbiota transplantation (FMT) gained favor among many physicians. In their thought-provoking opinion article, Nordin et al. argued that current evidence for FODMAPs-restricted diet in IBS is weak because most reported studies have a small sample size and lack blinding. Additionally, most of those studies focused on FODMAPs eliminations rather than provocations. Although a low FODMAPs diet could be part of a holistic IBS treatment, these complex dietary fibers are essential to a healthy diet and in line with official nutritional guidelines. Nordin et al. concluded that researchers should investigate the size effect in response to interventions and dose response in subjects with and without IBS in large double-blind dietary trials to establish more robust evidence. To further corroborate the utility of low FODMAPs diet, randomized controlled trials (RCTs) should be compared with the effectiveness of studies conducted in “real-world” settings.

Dietary modifications are among the most commonly used therapeutic interventions in IBS patients. Jeitler et al. conducted a multicenter RCT comparing Ayurvedic nutritional intervention vs. conventional dietary therapy, including low FODMAPs diet in IBS. They found that Ayurvedic and traditional diet therapy significantly reduced IBS symptom severity. A larger sample size and longer-term follow-up are warranted to corroborate these interesting findings.

The concept that gut dysbiosis may play a key role in the pathogenesis of IBS has led to adding FMT to our therapeutic armamentarium with different or occasionally conflicting outcomes (5). In their meta-analysis of FMT efficacy and safety in IBS, Samuthpongtorn et al. used a rigorous methodology to analyze nine RCTs. They found that FMT significantly improved symptom severity and health-related quality of life in the short but not the long term. However, global symptom improvement showed no significance. The improvement in clinical outcome with FMT for IBS may be ascribed to different routes of administration (colonoscopy-delivered FMT was superior), donor selection criteria, and donor microbiome profile.

The nowadays re-edited Rome IV criteria (Rome V are upcoming) proposed GI disorders as DGBI (instead of merely “functional”) because symptoms are generated by complex interactions among factors such as gut dysbiosis, altered visceral signaling, and disordered central nervous system processing (6). Patients with IBS commonly experience psychiatric disorders like depression and anxiety, regardless of the IBS subtype. In the American College of Gastroenterology clinical guidelines for IBS management (2), the expert panel suggested that physicians should consider gut-directed psychotherapy, including hypnotherapy, to treat global IBS symptoms.

In a qualitative community case study, Gerson et al. explored patients' experiences in virtual, group-based, gut-directed hypnotherapy (GDH) to treat various DGBIs, including IBS. The authors showed GI symptom improvement and wide acceptance of this group-based, remotely-delivered GDH. Thus, this treatment modality should be considered in gastroenterology practice as a single or add-on option in the conventional “step-up” strategy.

A rigorous meta-analysis of colorectal cancer (CRC) risk in patients with IBS by Wu et al. included six studies of 1,085,024 participants. Interestingly, the authors found that patients diagnosed with IBS before age 50 and those within the first year of IBS diagnosis have an increased risk for CRC. A more recent meta-analysis found a significant decrease in the prevalence of colorectal polyps and CRC in IBS patients compared to controls (7). In contrast, pooled CRC data did not reveal a significant reduction in CRC prevalence among IBS patients. However, current evidence does not support that IBS increases the risk for or protects against CRC. The early excess risk is probably attributable to misclassification resulting from overlapping symptoms rather than causality.

In summary, this Research Topic of articles brings together essential advancements in the field of IBS. However, IBS has been and likely will remain a challenging disease for most sufferers and physicians. The multifaceted pathophysiology and ill-defined drug targets make the goal of a “magic bullet” for IBS hard to achieve. Studies are warranted to further explore the pathophysiology of this complex disorder, the diagnosis durability, novel treatment options, and how these issues are intertwined.

## Author contributions

KJ drafted the manuscript. RD and DS provided critical revisions and approved the submitted manuscript. All authors have contributed substantially, directly, and intellectually to the work.
